# Brain Microstructural Damage as Potential Biomarker of Immune Cell‐Associated Neurotoxicity Syndrome

**DOI:** 10.1111/jon.70115

**Published:** 2026-01-16

**Authors:** Caterina Lapucci, Massimiliano Gambella, Emilio Cipriano, Anna Maria Raiola, Riccardo Varaldo, Anna Ghiso, M. Centanaro, E. Capello, A. Schenone, Lucio Castellan, Laura Barletta, Emanuele Angelucci, Matilde Inglese

**Affiliations:** ^1^ IRCCS Ospedale Policlinico San Martino Genoa Italy; ^2^ UO Ematologia e Terapie Cellulari IRCCS Ospedale Policlinico San Martino Genoa Italy; ^3^ Department of Neuroscience, Rehabilitation, Ophthalmology, Genetics, Maternal and Child Health (DINOGMI) University of Genoa Genoa Italy; ^4^ Anesthesiology and Intensive Care Unit IRCCS Ospedale Policlinico San Martino Genoa Italy

**Keywords:** chimeric antigen receptor–engineered T‐cell (CAR‐T), Cytokine Release Syndrome (CRS), diffusion, Immune Effector Cell–Associated Neurotoxicity Syndrome (ICANS), MRI

## Abstract

**Background and Purpose:**

Chimeric antigen receptor–engineered T‐cell (CAR‐T) therapy in hematological malignancies may be associated with severe complications, as Cytokine Release Syndrome (CRS) and Immune effector Cell‐Associated Neurotoxicity Syndrome (ICANS). The aim of the study is to investigate MRI‐derived macrostructural and microstructural features potentially able to identify patients at higher ICANS risk.

**Methods:**

Forty‐two patients treated with CAR‐T from October 2020 to June 2025 performed brain MRIs before CAR‐T administration, including diffusion‐weighted imaging. A general linear model was used to compare patients who developed ICANS, CRS, or neither at baseline in terms of MRI macro‐ and microstructural features. A binary logistic regression analysis was performed to evaluate the role of microstructural features in predicting the risk of developing ICANS.

**Results:**

Mean age 59.2 ± 13 years, 59.5% male; 21 (50%) patients received tisagenlecleucel, 21 (50%), axicabtagene ciloleucel or brexucabtagene autoleucel; 14 (33%) and 31 (73.8%) patients developed ICANS and CRS, respectively. At baseline MRI, fluid‐attenuated inversion recovery (FLAIR) white matter (WM) hyperintensities were detected in 41/42 (97.6%). No significant differences between patients who developed ICANS, CRS and neither both were observed in terms of FLAIR hyperintensities nor total brain volume at baseline. Fractional anisotropy extracted from FLAIR hyperintensities and WM areas without macroscopic abnormalities was a predictor of ICANS in the logistic regression model (*p* = 0.03 and 0.02, respectively).

**Conclusions:**

FLAIR hyperintensities and brain volume prior to CAR‐T were not informative, whereas the severity of WM microstructural (axonal) damage predicted ICANS risk. Greater axonal damage was associated with a higher likelihood of ICANS.

## 1 Introduction

Cytokine Release Syndrome (CRS) is a systemic inflammatory response that can occur after certain immunotherapies, most notably Chimeric Antigen Receptor–engineered (CAR) T‐cell therapy (CAR‐T) for hematological malignancies [1, 2]. CRS severity ranges from mild to life‐threatening [1, 2, 3] and is characterized by a marked release of pro‐inflammatory cytokines, such as IL‐6, IFN‐γ, and TNF‐α [2]. In some patients, CRS is complicated by Immune Effector Cell–Associated Neurotoxicity Syndrome (ICANS), although ICANS may also develop independently [1, 2, 3]. Clinical manifestations of ICANS include headache, confusion, seizures, cerebral edema, and coma [3]. A cytokine‐mediated inflammatory process affecting the central nervous system (CNS) is thought to underlie ICANS pathophysiology [2]. The prognosis of ICANS is strongly influenced by prompt initiation of treatment; therefore, early identification of patients at high risk is critical [4, 5, 6, 7, 8]. Previous studies have suggested potential associations between ICANS risk and factors, such as older age, pre‐existing (including neurological) conditions, CAR‐T construct type, and baseline inflammatory markers (C‐reactive protein, ferritin, thrombocytopenia, d‐dimer, fibrinogen, and lactate dehydrogenase [LDH])—often with conflicting results [1, 6, 7, 8, 9, 10, 11]. To date, no clear neuroradiological predictors of ICANS development, following CAR‐T therapy, have been established. Indeed, only a few studies, based on conventional MRI images, investigated baseline MRI findings prior to CAR‐T administration, most often with no conclusive results [6, 8]. In this scenario, advanced MRI techniques may help provide an in vivo characterization of microstructural damage within the brain tissue. Diffusion tensor imaging (DTI) has been the first diffusion method used to study microstructural features of the CNS as a whole and within specific voxels in demyelinating and non‐demyelinating diseases [12]. Among DTI‐derived metrics, fractional anisotropy (FA) provides information about microstructural integrity (i.e., axonal damage), whereas mean diffusivity (MD) suggests the amount of free diffusion due to tissue degeneration or edema [12]. Used together, FA and MD provide complementary insights into white matter (WM) microstructure and pathology. From a pathophysiological perspective, blood–brain barrier (BBB) impairment with endothelial activation [6], reduced microstructural resilience to cytokine‐mediated injury, and network‐level functional susceptibility [13] may be pathophysiologically related to WM microstructural damage and, thus, involved in ICANS development and explain ICANS clinical features. The aim of this study was to investigate whether MRI‐derived macrostructural and microstructural features can identify patients at increased risk of developing ICANS after CAR‐T therapy.

## 2 Methods

The analysis was conducted within a translational study approved by the Ethics Committee of IRCCS Ospedale Policlinico San Martino (Genoa) (CE 12742), conducted in accordance with the ethical principles of the Declaration of Helsinki. All participants provided consent to use their medical history for publication.

In this prospective study, 42 consecutive patients (age range at CAR‐T administration: 32–79 years) treated with CAR‐T from October 2020 to June 2025 at IRCCS Ospedale Policlinico San Martino in Genoa performed brain MRI (1.5T Siemens Avanto MRI scanner) before CAR‐T administration with the following acquisition protocol: 3‐dimensional (3D) sagittal T2‐fluid‐attenuated inversion recovery (FLAIR) (repetition time [TR] = 4500 ms, inversion time [TI] = 1800 ms, echo time [TE] = 400 ms, flip angle = 120°, voxel size = 1 × 1× 1 mm^3^); 3D sagittal T1‐magnetization prepared rapid gradient‐echo (MPRAGE) (TR = 2000 ms, TE = 3.4 ms, TI = 1100 ms, flip angle = 12°, voxel size = 1 × 1 × 1 mm^3^) before and after gadolinium administration; 2D echo‐planar‐imaging (EPI) diffusion MRI (dMRI) sequence with 60 directions and *b* value = 1000 s/mm^2^ (TR = 7500 ms, TE = 84 ms, flip angle = 90°, and voxel size = 3 × 3× 3 mm^3^).

For all patients, demographical and clinical data (CAR‐T construct, response to treatment), LDH levels, Eastern Cooperative Oncology Group (ECOG score), disease stage, bulky feature, and extra‐nodal involvement were achieved. CRS and ICANS were classified according to ASTCT consensus grading [14] and treated in accordance with European Hematology Association/European Society for Bone and Marrow Transplantation (EHA/EBMT) guidelines [15]. Patients, who developed both CRS and later ICANS, were considered ICANS patients.

### 2.1 MRI Analysis

We defined “FLAIR hyperintensity” any macroscopic WM abnormality regardless of its etiology (vascular‐origin changes, such as moderate to severe leukopathy, chronic post‐stroke lesions, radiation‐related changes, and hematological involvement) and “WM areas without macroscopic abnormalities” the brain parenchymal areas with no detectable hyperintensities or gadolinium enhancement on FLAIR and T1‐weighted images, respectively.

WM FLAIR hyperintensities and T1 hypointensities were manually segmented by using Jim software v.7 (Xinapse Systems Ltd, Northants, UK, http://www.xinapse.com) by a clinician expert in neuroimaging (C.L.). The FLAIR hyperintensities were then rigidly registered to T1 space with FSL [16] to compute the unremarkable WM by subtracting the lesions from the WM mask obtained via CAT12 [17]. Additionally, whole brain volume was calculated using SIENAX [16].

dMRI data analysis was conducted using MRtrix3 [18]. The preprocessing steps included denoising and correction of image artifacts caused by involuntary head motion and intensity inhomogeneities. The T1 images were then rigidly registered to dMRI space with FSL [16]. DTI metrics, including FA and MD, were then extracted within both FLAIR hyperintensities and WM areas without macroscopic abnormalities. All these analyses were performed by a physicist expert in imaging processing (E.C.).

### 2.2 Statistical Analysis

Demographic and clinical variables were converted in a nominal feature (altered “yes/no”). Demographic and clinical data of patients who developed CRS, ICANS, or neither were compared across the three groups to evaluate possible confounding variables. A general linear model (GLM) adjusted for confounding variables was used to compare, in terms of microstructural features, patients who developed ICANS, CRS, or neither after CAR‐T treatment. A binary logistic regression analysis was performed to evaluate the role of microstructural features at baseline MRI in predicting the risk of developing ICANS. Statistical analysis was performed using SPSS software version 20. The results were considered statistically significant if the corresponding *p* value was inferior to 0.05. A trend was considered if the *p* value was inferior to 0.1.

## 3 Results

### 3.1 Study Population

Forty‐two (*n* = 42) patients [(mean age 59.2 ± 13 years, 59.5% male, *n* = 2 affected by follicular lymphoma, *n* = 5 affected by mantellar lymphoma (MLC), and *n* = 35 affected by diffuse large B‐cell lymphoma (DLBCL)]; 21 (50%) patients received tisagenlecleucel (tisa‐cel), and 21 (50%) received axicabtagene ciloleucel (axi‐cel) or brexucabtagene autoleucel (brexu‐cel). Total patients who developed CRS were 31 (73.8%); 14 (33%) developed isolated CRS, and 17 (40%) patients developed ICANS following CRS (ICANS group). Mean age for ICANS group was 59 ± 12.6 years (range: 32–79 years), for CRS group was 56.5 ± 14 years (range: 32–75.1 years), and for no complications group was 63.9 ± 11.7 years (range: 33.8–75 years).

All patients received standard therapy for CRS and ICANS management in accordance with EBMT/JACIE/EHA guidelines for patients receiving CAR‐T cells [15]. In particular, patients received up to four doses of tocilizumab for CRS and dexamethasone for CRS and/or ICANS. Dexamethasone dose was modulated in accordance with the severity of CRS and ICANS, in accordance with the abovementioned guidelines [14, 15].

The median number of chemotherapy systemic lines before CAR‐T therapy per patient was two (range: 1–4). Two patients received second‐line treatment with axi‐cel; one patient received brain radiotherapy in advance of CAR‐T infusion due to CNS hematological involvement (cranial nerve involvement). All other patients showed an unremarkable neurological examination before CAR‐T treatment initiation.

Isolated CRS was of Grade 4 in 2 patients (4.8%), Grade 3 in 1 patient (2.4%), Grade 2 in 4 patients (9.5%), and Grade 1 in 10 patients (23.8%). As concerns total CRS, CRS was of Grade 4 in 11 patients (26.2%), Grade 3 in 6 patients (14.3%), Grade 2 in 4 patients (9.5%), and Grade 1 in 10 patients (23.8%).

ICANS was of Grade 4 in 3 patients (7.1%), Grade 3 in 6 patients (14.3%), Grade 2 in 1 patient (2.4%), and Grade 1 in 4 patients (9.5%).

Demographic and clinical features of our patients’ cohort are detailed in Table 1.

**TABLE 1:** Demographic and clinical characteristics of patients treated with CAR‐T.

Total no. of patients (*n*)	42
Age (years)	59.2 ± 13 range (32–79)
Male (%)	59.5
Haematological malignancy	
Follicular lymphoma, *n* (%)	2 (4.8)
Diffuse large B‐cell lymphoma, *n* (%)	35 (83.3)
Mantellar lymphoma, *n* (%)	5 (11.9)
CAR‐T construct	
tisagenlecleucel, *n* (patients) (%)	21 (50)
axicabtagene ciloleucel/brexucabtagene autoleucel, *n* (patients) (%)	21 (50)
Clinical and laboratory features	
Response to treatment, absent, *n* (%)	18 (42.9)
LDH increase, *n* (%)	23 (54.8)
ECOG score >2, *n* (%) disease Stages III–IV, *n* (%)	5 (11.9) 27 (64.3)
Bulky feature, *n* (%)	11 (26.2)
Extra‐nodal involvement, *n* (%)	20 (47.6)
Isolated CRS, *n* (patients) (%)	17 (40.5)
Grade 1	10 (23.8)
Grade 2	4 (9.5)
Grade 3	1 (2.4)
Grade 4	2 (4.8)
Total CRS, *n* (patients) (%)	31 (73.8)
Grade 1	10 (23.8)
Grades 2 and 3	4 (9.5) 6 (14.3)
Grade 4	11 (26.2)
ICANS, *n* (patients) (%)	14 (33.3)
Grade 1	4 (9.5)
Grade 2	1 (2.4)
Grade 3	6 (14.3)
Grade 4	3 (7.1)

*Note*: All the data represent mean ± standard deviation unless otherwise indicated.

Abbreviations: CAR‐T = chimeric antigen receptor–engineered T‐cell therapy; CRS = Cytokine Release Syndrome; ECOG = Eastern Cooperative Oncology Group; ICANS = immune effector cell‐associated neurotoxicity syndrome; LDH = lactate dehydrogenases; *n* = number; SD = standard deviation.

No significant differences were observed in terms of response to treatment, LDH levels, ECOG score, disease stage, bulky feature, extra‐nodal involvement, and CAR‐T construct between patients who developed ICANS, CRS, or neither. Thus, none of these variables were used as covariate in the GLM and regression analysis. Furthermore, when stratifying patients by CAR‐T product, baseline characteristics such as age, sex, and relevant clinical variables (response to treatment, LDH levels, ECOG score, disease stage, bulky feature, and extra‐nodal involvement) did not significantly differ across the subgroups.

Age, gender, and FLAIR hyperintensities volume were used as covariate as appropriate for microstructural analysis.

### 3.2 MRI Features

### 3.3 Conventional MRI Features

- FLAIR WM hyperintensities (i.e., chronic vascular, post‐stroke, and radiation related) were detected in 41/42 (97.6%) patients.
- 1/42 patients showed brain macrostructural features suggestive of active hematological involvement (multiple cranial nerve enhancement; no leptomeningeal nor parenchymal enhancement was otherwise observed in our patients’ cohort).

### 3.4 Advanced MRI Features

### 3.5 FLAIR Lesion Load and Total Brain Volume

After adjusting for age and gender, we did not observe significant differences between patients who developed ICANS, CRS, or neither both in terms of FLAIR hyperintensities (1.0 ± 2.9 vs. 7.9 ± 2.6 vs. 0.07 ± 3.3 mL, *F*(2, 24.4) = 1.51, *p* = 0.241) and total brain volume (1385.5 ± 156.9 vs. 1393.2 ± 129.3 vs. 1424.1 ± 159.3 mL, *F*(2, 23.7 = 0.558, *p* = 0.580).

### 3.6 DTI Analysis

- FLAIR WM hyperintensities: ICANS (*n* = 14) versus CRS (*n* = 17) versus no complications (*n* = 11), after adjusting for age, gender, and FLAIR hyperintensities volume: Mean (±SD) FA was lower in ICANS with respect to CRS and patients without complications (0.26 ± 0.06 vs. 0.29 ± 0.02 vs. 0.32 ± 0.09, respectively; *p* = 0.013 for ICANS vs. no complications). FA was lower in patients with ICANS Grades 3–4 with respect to that of patients with ICANS Grades 1–2 (0.237 ± 0.08 vs. 0.286 ± 0.008, respectively). No differences were observed in terms of MD metric [1.1 ± 0.2 (×10^−3^ mm^2^/s) vs. 1.0 ± 0.1 (×10^−3^ mm^2^/s) vs. 1.1 ± 0.1 (×10^−3^ mm^2^/s), respectively].
- WM areas without macroscopic abnormalities: ICANS (*n* = 14) versus CRS (*n* = 17) versus no complications (*n* = 11), after adjusting for age, gender, and FLAIR hyperintensities volume: Mean (±SD) FA was lower in ICANS with respect to patients with CRS and patients without complications (0.35 ± 0.02 vs. 0.37 ± 0.02 vs. 0.38 ± 0.03, respectively; *p* = 0.014 for ICANS vs. CRS and *p* = 0.005 for ICANS vs. no complications). FA was lower in patients with ICANS Grades 3–4 with respect to that of patients with ICANS Grades 1–2 (0.341 ± 0.02 vs. 0.369 ± 0.02, respectively). No differences were observed in terms of MD metric [0.78 ± 0.03 (×10^−3^ mm^2^/s) vs. 0.78 ± 0.02 (×10^−3^ mm^2^]/s) vs. 0.77 ± 0.03 (×10^−3^ mm^2^/s), respectively].

**FIGURE 1:**
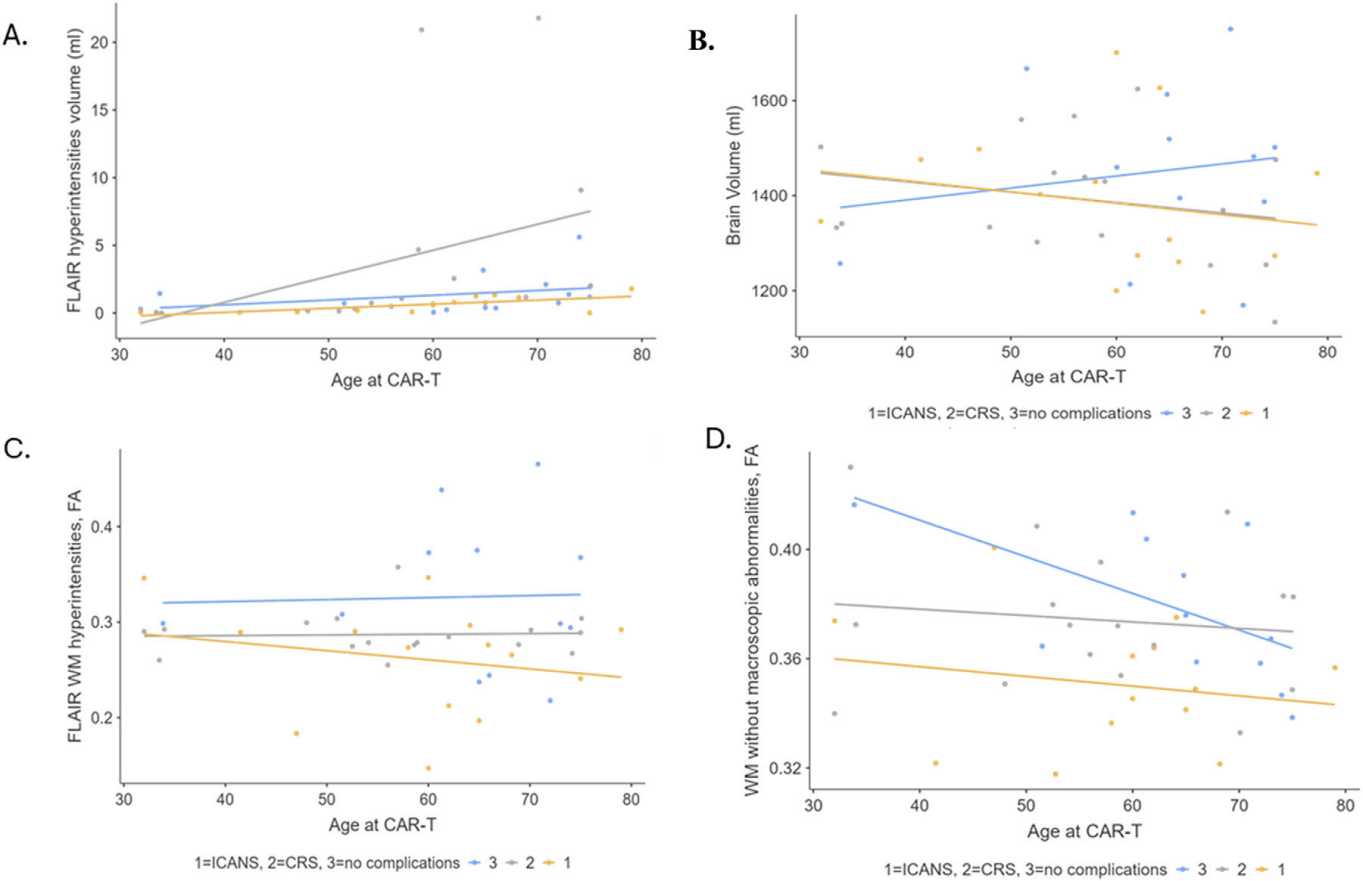
Correlations (scatter plots) between age at chimeric antigen receptor‐engineered T‐cell (CAR‐T) administration and fluid attenuated inversion recovery (FLAIR) hyperintensities volume (A), brain volume (B), FLAIR white matter (WM) hyperintensities fractional anisotropy (FA) (C), and WM without macroscopic abnormalities FA (D).

No correlations were found between age at CAR‐T and FLAIR hyperintensities volume, age at CAR‐T and brain volume, age at CAR‐T and FLAIR WM hyperintensities FA, and age at CAR‐T and WM without abnormalities FA (Figure 1A–D).

FA extracted from FLAIR WM hyperintensities and WM areas without macroscopic abnormalities was a significant predictor of ICANS after CAR‐T in the logistic regression model (*p* = 0.03, OR = 0.01 95% CI [0–0.19] and *p* = 0.02, OR = 0.01 95%, and CI [0–0.02], respectively), with lower FA values associated with higher risk of ICANS development.

**FIGURE 2:**
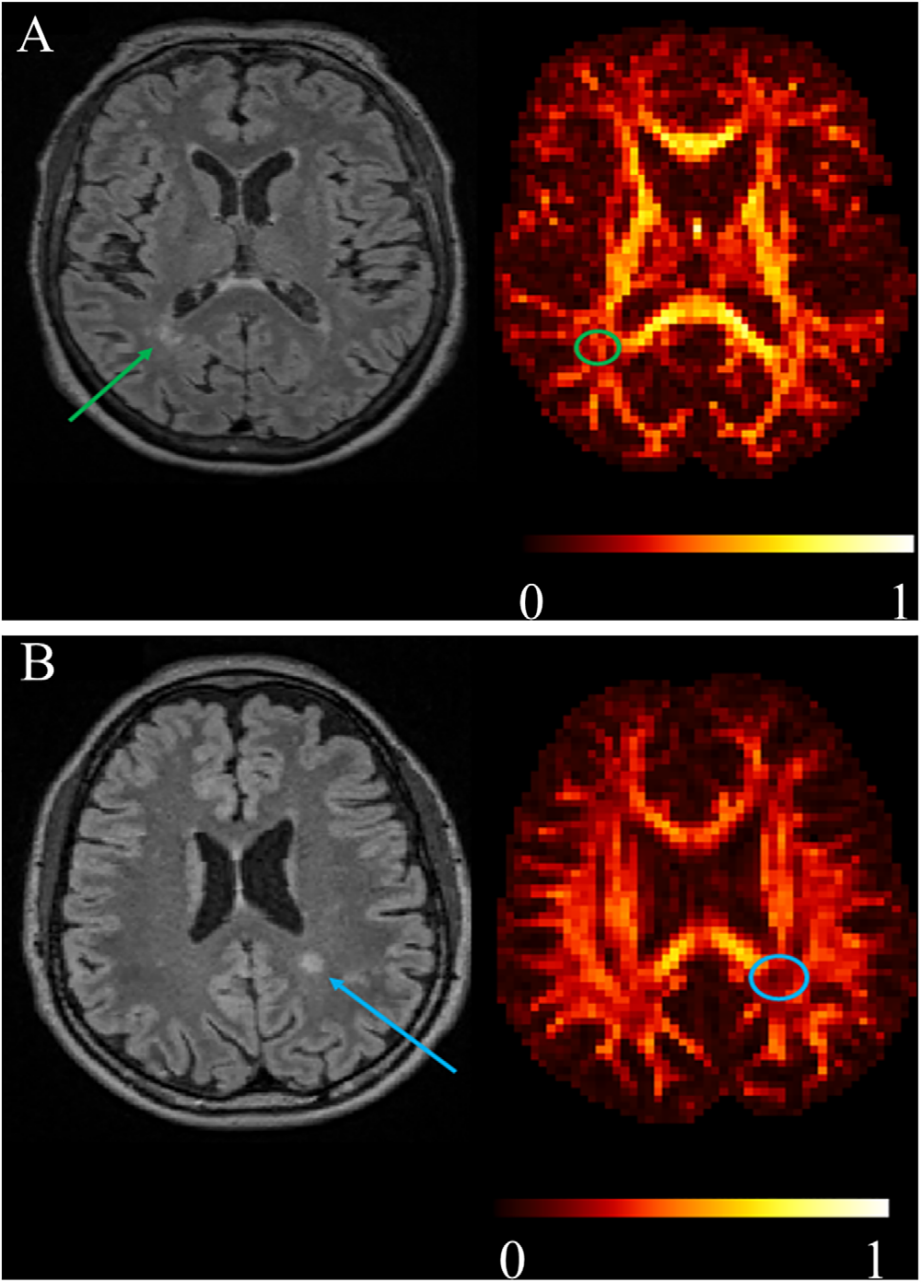
(A) Selected fluid attenuated inversion recovery (FLAIR) images and fractional anisotropy (FA) maps from a 67‐year‐old patient who did not exhibit any chimeric antigen receptor‐engineered T‐cell (CAR‐T)‐related complication. FLAIR white matter hyperintensity (green arrow) shows higher FA value (less severe axonal damage) (green circle) with respect to patient illustrated in Figure 1. (B) Selected FLAIR images and FA maps from a 51‐year‐old patient who developed Immune effector Cell‐Associated Neurotoxicity Syndrome (ICANS) after Car‐T treatment. FLAIR white matter (hyperintensity (blue arrow) shows lower FA value (more severe axonal damage) (blue circle) with respect to patient illustrated in Figure 1A.

Baseline (before CAR‐T treatment) MRI features of our patients’ cohort are detailed in Table 2. Representative cases are reported in Figure 2A,B.

**TABLE 2:** Baseline (before chimeric antigen receptor‐engineered T‐cell [CAR‐T] treatment) MRI features.

	ICANS (*n* = 14)	CRS (*n* = 17)	No complications (*n* = 11)	*p* value
FLAIR hyperintensities volume (mL)	1.0 ± 2.9	7.9 ± 2.6	0.07 ± 3.3	0.241–
Brain volume (mL)	1385.5 ± 156.9	1393.2 ± 129.3	1424.1 ± 159.3	0.580
FLAIR WM hyperintensities, FA	0.26 ± 0.06	0.29 ± 0.02	0.32 ± 0.09	0.013^*^ ICANS vs. no complications
FLAIR WM hyperintensities, MD (×10^−3^ mm^2^/s)	1.1 ± 0.2	1.0 ± 0.1	1.1 ± 0.1	0.671
WM without macroscopic abnormalities, FA	0.35 ± 0.02	0.37 ± 0.02	0.38 ± 0.03	0.014^*^ ICANS vs. CRS 0.005^*^ ICANS vs. no complications
WM without macroscopic abnormalities, MD (×10^−3^ mm^2^/s)	0.78 ± 0.03	0.78 ± 0.02	0.77 ± 0.03	0.542

*Note*: All the data represent mean ± standard deviation unless otherwise indicated.

Abbreviations: CRS = Cytokine Release Syndrome; FA = fractional anisotropy; FLAIR = fluid attenuated inversion recovery; ICANS = Immune Effector Cell‐Associated Neurotoxicity Syndrome; MD = mean diffusivity; *n* = number; NAWM = normal appearing white matter; WM = white matter.

* *p* value < 0.05.

## 4 Discussion

In the present study, we focused on MRI features obtained prior to CAR‐T treatment by conventional and advanced DTI analysis to identify neuroradiological biomarkers able to identify patients at higher risk of developing ICANS. Indeed, whereas several studies have investigated MRI [6, 8] and PET [19] findings during ICANS, to our knowledge no neuroimaging biomarkers have been evaluated for predicting the risk of ICANS development.

First, we analyzed potentially confounding clinical variables. Notably, no differences were found in treatment response, LDH levels, ECOG score, disease stage, bulky disease, extranodal involvement, or CAR‐T construct. These findings are consistent with the current literature, in which various studies have identified clinical and laboratory predictors of CRS and ICANS—often with contradictory results [3, 4, 5].

Regarding conventional imaging, only one patient in our cohort showed radiological evidence of CNS hematological involvement (multiple cranial nerve enhancement), for which brain irradiation was administered in addition to CAR‐T therapy. No patients exhibited MRI features suggestive of parenchymal lymphomatous involvement. Most patients (97.6%) presented with focal and/or diffuse WM hyperintensities on FLAIR images, likely attributable to vascular causes (e.g., age‐related small vessel disease) or iatrogenic factors (e.g., prior brain irradiation, chemotherapy).

Only a few case series described baseline MRI findings prior to CAR‐T administration, most often reporting unremarkable scans or nonspecific periventricular WM hyperintensities as the most common observations [6, 8]. Gust et al. reported associations between MRI abnormalities [11] and comorbidities, such as seizures or prior CNS toxicity [6]; however, baseline imaging was not available for all patients, and causal relationships were not specifically examined, limiting generalizability. In a recent study, pre‐existing neurological injury—defined as previous neurological events and pre‐CAR‐T MRI abnormalities—was not associated with an increased risk of ICANS [20].

Consistent with these findings, we did not observe significant differences between patients who subsequently developed ICANS and CRS and those who did not, in terms of either FLAIR hyperintensities or total brain volume. Thus, neither the macroscopic extent of focal WM hyperintense areas nor the severity of brain atrophy before CAR‐T therapy appeared to influence the likelihood of developing ICANS.

Our findings appear to contrast with current recommendations, which state that the presence of “pre‐existing and/or active neurological disorders, especially those with uncontrolled or untreated CNS involvement,” represents a contraindication to CAR‐T therapy due to the high risk of associated complications. However, this definition is broad and lacks specificity. Similarly, although the term *active* is intuitive when referring to lymphomatous CNS involvement, its meaning is less clear for other neurological comorbidities, such as vascular or inflammatory conditions. Moreover, it is well known that FLAIR hyperintensities may reflect various pathological substrates—including gliosis, demyelination, and remyelination—making them highly nonspecific. Therefore, in our view, the sole MRI evidence of macroscopic WM abnormalities should not be considered a sufficient criterion for stratifying patients in terms of ICANS risk.

Conversely, we found that FA values—both within WM FLAIR hyperintense areas and in WM regions without macroscopic abnormalities—before CAR‐T therapy were significantly lower in patients who later developed ICANS compared with those who did not, with the lowest values observed in the ICANS group. Moreover, FA in both WM compartments was predictive of ICANS development. Given that DTI‐derived FA reflects in vivo WM integrity, these findings suggest that greater axonal loss is associated with an increased risk of ICANS after CAR‐T cell therapy. From a pathophysiological perspective, endothelial activation—implicating BBB impairment and concomitant neuronal damage—has been proposed in the pathogenesis of ICANS [6]. In this context, we speculate that focal and diffuse WM integrity loss (expressed by low FA values), secondary to vascular comorbidities and/or the toxicity of prior hematological treatments, may contribute to BBB disruption and, consequently, to the cytokine cascade that underlies the immunological hallmarks of ICANS. Furthermore, consistent with our findings, recent evidence suggests that elevated serum light chain neurofilaments (NfL) levels—reflecting pre‐existing or latent neuroaxonal injury—may serve as a predictive biomarker for ICANS development and severity, and for improving patient monitoring after CAR T‐cell infusion [21, 22]. Furthermore, WM characterized by reduced FA might be more susceptible to secondary injury in inflammatory states (as in high‐grade CRS), due to pre‐existing impairment of axonal membranes. This phenomenon may reduce the threshold for neurotoxic cascades, including excitotoxicity, oxidative stress, and microglial activation, as observed during and after COVID‐19 infection, a condition where, similarly to ICANS after CAR‐T, an inflammatory cascade mediated by cytokine storm may finally result in neurological involvement [23]. Finally, WM microstructural abnormalities revealed by low FA values may disproportionately affect large‐scale functional networks [13] implicated in attention, arousal, and executive functioning—domains frequently impaired in ICANS [1].

This exploratory study has several limitations, primarily the small sample size and the relatively low number of ICANS cases.

In conclusion, the presence of macroscopic WM abnormalities prior to CAR‐T treatment did not predict the risk of developing ICANS and, therefore, would not be useful for patient stratification or for guiding therapeutic decisions. In contrast, the severity of pre‐existing microstructural parenchymal damage—particularly axonal injury—may serve as a neuroradiological biomarker to identify patients at higher risk of developing ICANS following CAR‐T cell therapy.

Further studies in larger patient cohorts are warranted to validate these findings.

## Funding

This work was developed thanks to the support from #NEXTGENERATIONEU (NGEU) of the Ministry of University and Research (MUR), National Recovery and Resilience Plan (NRRP), project (1) MNESYS (PE0000006)—a multiscale integrated approach to the study of the nervous system in health and disease (DN. 1553 11.10.2022) and (2) Fit4med rob.

## Conflicts of Interest

Caterina Lapucci reported no conflicting interests. Massimiliano Gambella reported no conflicting interests related to the study. Emilio Cipriano reported no conflicting interests related to the study. Anna Maria Raiola and Riccardo Varaldo reported no conflicting interests related to the study. Anna Ghiso reported no conflicting interests related to the study. Monica Centanaro reported no conflicting interests related to the study. E. Capello reported no conflicting interests related to the study. A. Schenone reported no conflicting interests related to the study. Lucio Castellan reported no conflicting interests related to the study. Laura Barletta reported no conflicting interests related to the study. Emanuele Angelucci declared DMC chair for Vertex and BMS, DMC member per Vifor, Adv board per Novartis e Regeneron, Speaker per BMS, Contract per Sanofi. Matilde Inglese reported no conflicting interests related to the study.
